# Successful Treatment of Autoimmune Hemolytic Anemia Concomitant with Proliferation of Epstein-Barr Virus in a Post-Heart Transplant Patient

**DOI:** 10.3390/hematolrep14030036

**Published:** 2022-08-17

**Authors:** Dan Ran Castillo, Parthiv Sheth, Kevin Nishino, Wesley Tait Stevens, Anthony Nguyen, Alberto Romagnolo, Hamid Mirshahidi

**Affiliations:** 1 Hematology/Oncology Department, Loma Linda University Medical Center, Loma Linda, CA 92354, USA; 2 Department of Internal Medicine, Loma Linda University Medical Center, Loma Linda, CA 92354, USA; 3 Department of Pathology, Loma Linda University Medical Center, Loma Linda, CA 92354, USA; 4 Moores Cancer Center, UCSD, San Diego, CA 92037, USA

**Keywords:** EBV, heart transplantation, AIHA, rituximab

## Abstract

Autoimmune hemolytic anemia (AIHA) is a rare complication following heart transplantation and has been attributed to several etiologies including infections, immunosuppressive medications, and post-transplant lymphoproliferative disorders. We report a 23-year-old male presenting 22 years after heart transplantation with severe AIHA. Laboratory findings were notable for positive IgG autoantibody against RBCs and high titer Epstein-Barr virus (EBV) viremia. Shortly after the first unit of irradiated RBC transfusion and high dose steroids, the patient developed acute dyspnea and hypoxia requiring intubation. Further workup demonstrated that the patient had Methicillin-sensitive Staphylococcus aureus (MSSA) pneumonia (PNA) and bacteremia, requiring antibiotics. Patient was subsequently treated with high-dose steroids, IVIG, as well as rituximab. Following treatment, the patient was successfully extubated and eventually showed complete resolution of the anemia. This case is novel as it represents AIHA likely secondary to EBV viremia in a post-cardiac transplant patient complicated by a severe transfusion reaction. In this circumstance, rituximab in conjunction with standard of care remains an effective treatment of choice.

## 1. Introduction

AIHA, an acquired hemolysis characterized by the presence of specific autoantibodies against components of red blood cells, affects roughly 1 to 3 per 100,000 people each year [1]. Typically, diagnosis first surrounds identifying hemolysis with a normocytic or macrocytic anemia, elevated reticulocyte count, elevated unconjugated bilirubin, reduced haptoglobin and a peripheral blood smear with polychromasia [2]. Further studies such the direct antiglobulin test (DAT) which reveals IgG and complement binding to erythrocytes can subsequently confirm whether this hemolysis is secondary to immune processes [3]. A diagnosis of AIHA can thus be made once other alternatives such as a delayed transfusion reaction, alloimmune hemolysis following organ or allogeneic stem cell transplantation, and drug-immune hemolysis have been excluded.

Epstein-Barr virus (EBV) infection should be considered in any hemolytic anemia associated with hepatic dysfunction, especially when a direct antiglobulin test is positive for C3d. In these cases, a course of corticosteroids seems safe and may be beneficial. infection is associated with several hematologic complications that are typically mild and self-limited. Among them, the frequency of clinically significant hemolysis in EBV infections is estimated to be low, and most of these cases are associated with cold agglutinins 9. EBV infection should be considered in any hemolytic anemia associated with hepatic dysfunction, especially when a direct antiglobulin test is positive for C3d. In these cases, a course of corticosteroids seems safe and may be beneficial. Virus infection should be considered in any hemolytic anemia associated with hepatic dysfunction, especially when a direct antiglobulin test is positive for C3d. In these cases, a course of corticosteroids seems beneficial.

Treatment of primary AIHA usually surrounds therapies directed at immunosuppression. Although no consensus guidelines exist, initial treatment of AIHA is primarily with prednisolone or prednisone [4]. Patients who do not respond to treatment with corticosteroids should subsequently be treated with azathioprine or mycophenolate mofetil. In refractory cases, rituximab is indicated. Blood transfusion should be readily utilized in patients with hypoxic anemia.

Tacrolimus is a common immunosuppressant used in patients after solid organ transplantation to prevent rejection. However, it is well-documented that acute hemolytic anemia is a side-effect of this therapy. In this report, we describe a cardiac transplant patient who developed autoimmune hemolytic anemia while undergoing treatment with tacrolimus.

## 2. Case Report

We describe a 23-year-old male with a past medical history of heart transplantation at age 1 for hypoplastic left heart syndrome on prednisone and tacrolimus and post-transplant lymphoproliferative disorder of bowel at age 10. Other home medications included aspirin, enalapril, pravastatin, and prophylactic amoxicillin. Per the patient’s mother, he was in his usual state of health until one week prior when they tried going for a walk but could not since the patient felt too weak. He later experienced worsening fatigue, dizziness, headache, and jaundice. He denied any fevers, chills, vomiting, cough, shortness of breath, diarrhea, bloody stool, or new rashes. He additionally reported no sick contacts or recent travel.

Upon presentation to the emergency department, physical examination was significant for scleral icterus, jaundice, tachycardia, capillary refill >3 s, and tenderness in the RUQ with hepatomegaly. Complete blood count showed a hemoglobin of 3.1 g/dL (significantly decreased from 16.3 g/dL one month prior), WBC 13,160/µL, platelet 54,000/µL and reticulocyte count >23.0% (absolute count 321/µL). Further studies revealed total bilirubin 6.9 mg/dL, indirect bilirubin 6.3 mg/dL, haptoglobin <10 mg/dL, and lactate dehydrogenase (LDH) 356 U/L, high serum bilirubin makes hemolysis highly suspicious. There was no evidence of hemoglobinuria or bilirubinuria. Direct and indirect Coombs tests were positive (direct Coombs test: +++, indirect Coombs test: +++). Red blood cell-associated IgG (RBC-IgG) was elevated to 477 molecules per one RBC (normal range; 20–46 molecules per one RBC). Antibody identification was anti-RhC+e antigen. Peripheral smear was reviewed and revealed spherocytes, polychromatic cells, and nucleated erythrocytes with no schistocytes visualized, decreasing suspicion for microangiopathic hemolytic anemia. Of note, EBV DNA by PCR noted 20,500 copies/mL.

Given findings highly suspicious for autoimmune hemolytic anemia (AIHA) twenty-two years after transplantation, the patient was transfused with irradiated pRBC and started on IV methylprednisolone 1 mg/kg daily. Tacrolimus was also held. The blood bank did identify a strong autoantibody. Due to the severity of anemia, a single unit transfusion was attempted. However, shortly after transfusion of the unit, rapid response was called for shortness of breath and hypoxia, eventually resulting in intubation. Patient was started on intravenous immunoglobin (IVIG) 1 g/kg for two days, epogen, folic acid, iron sucrose, and cyclosporine. Additionally, the patient was given one dose of rituximab 700 mg. Over this period, the patient was additionally treated for MSSA pneumonia with bacteremia and acute kidney injury.

Patient was eventually extubated on day 7 of admission and continued receiving rituximab weekly. He was continued on epogen, folic acid, and solumedrol daily. Hgb remained low (~4.0 g/dL) for most of the hospital course. However, on day 17 of admission, the patient’s Hgb began to show improvement. He was ultimately discharged on day 18 with Hgb at 7.5 g/dL.

**Figure 1:**
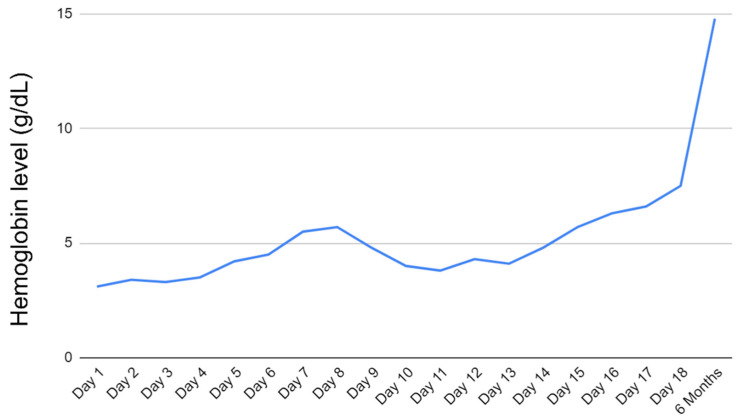
The trend and improvement of hemoglobin at pre- and post-treatment.

Upon outpatient follow-up, the patient reported feeling well with mild fluid retention improving on furosemide. He was started on a prednisone taper beginning at 70 mg and decreased by 10 mg weekly. Including rituximab infusions given as inpatient, the patient received a total of 4 cycles. Additionally, his immunosuppression was transitioned from tacrolimus to cyclosporine by his cardiologist. Hemoglobin approximately 5 months after discharge was 14.8 g/dL. The trend and improvement of hemoglobin are shown in Figure 1.

## 3. Discussion

Autoimmune hemolytic anemia has previously been seen in patients following solid organ transplant and attributed to multiple etiologies including infectious causes such as Epstein-Barr viremia, immunosuppressive medications such as tacrolimus, and post-transplant lymphoproliferative disorder [5]. In one retrospective single-centered study, it was reported that 5 out of 103 (4.9%) pediatric patients who received heart transplant developed autoimmune cytopenia including autoimmune hemolytic anemia. Another retrospective single-centered study demonstrated that 3 out of 188 patients (0.016%) developed autoimmune hemolytic anemia following heart transplant [6].

In this case, the patient developed severe autoimmune hemolysis approximately 22 years following cardiac transplantation. The etiology of this is unclear as the patient has several risk factors described above including multiple prior blood transfusions, EBV viremia, tacrolimus use, and history of post-transplant lymphoproliferative disorder. Initially, the patient was thought to have developed hemolytic anemia due to tacrolimus. It has been previously well-documented that post-transplant patients taking immunosuppressive medications such as tacrolimus may induce hemolytic reactions [7]. The two mechanisms which have been described to cause these presentations include drug-induced hemolysis as well as alloimmune hemolysis because of lymphocytes from donor allograft [7]. However, these adverse events typically occur within 1 year of transplantation and would be highly unusual to cause this patient’s hemolysis who is over 20 years removed from transplantation.

Additionally, the patient was noted to be EBV positive with significant viremia. Primary EBV infection is associated with AIHA in 0.1 to 3% of cases and the mechanism by which this occurs is not well-understood [8]. Latent EBV infection may also exacerbate an existing warm AIHA. In these cases, EBV is typically noted to cause erythropoietic failure and subsequently low reticulocyte counts. EBV is classically associated with cold agglutin AIHA [8,9]. The cold-agglutinin test result in this case was negative.

While this would be inconsistent with the laboratory findings in the patient we present, the noted increase in hemoglobin and decreased viremia possibly indicates a decrease in EBV infected cells in response to rituximab therapy [10].

This case demonstrates a novel presentation and treatment of autoimmune hemolytic anemia in a post cardiac transplant patient where continued blood transfusion support was not possible following a severe transfusion reaction. While the precipitating cause is not definitively known, eventual improvement following immunomodulatory support with rituximab further supports its use in transplant patients with EBV viremia.

## Data Availability

Not applicable.
